# Development of an Immunoperoxidase Monolayer Assay for the Detection of Antibodies against Peste des Petits Ruminants Virus Based on BHK-21 Cell Line Stably Expressing the Goat Signaling Lymphocyte Activation Molecule

**DOI:** 10.1371/journal.pone.0165088

**Published:** 2016-10-21

**Authors:** Jialin Zhang, Wenxing Liu, Weiye Chen, Cuicui Li, Meimei Xie, Zhigao Bu

**Affiliations:** National Key Laboratory of Veterinary Biotechnology, Harbin Veterinary Research Institute, Chinese Academy of Agricultural Sciences (CAAS), Harbin, China; Indian Institute of Science, INDIA

## Abstract

From 2013 to 2015, peste des petits ruminants (PPR) broke out in more than half of the provinces of China; thus, the application and development of diagnostic methods are very important for the control of PPR. Here, an immunoperoxidase monolayer assay (IPMA) was developed to detect antibodies against PPR. However, during IPMA development, we found that Vero cells were not the appropriate choice because staining results were not easily observed. Therefore, we first established a baby hamster kidney-goat signaling lymphocyte activation molecule (BHK-SLAM) cell line that could stably express goat SLAM for at least 20 generations. Compared with Vero cells, the PPR-mediated cytopathic effect occurred earlier in BHK-SLAM cells, and large syncytia appeared after virus infection. Based on this cell line and recombinant PPR virus expressing the green fluorescent protein (GFP) (rPPRV-GFP), an IPMA for PPR diagnosis was developed. One hundred and ninety-eight PPR serum samples from goats or sheep were tested by the IPMA and virus neutralization test (VNT). Compared with the VNT, the sensitivity and specificity of the IPMA were 91% and 100%, respectively, and the coincidence rate of the two methods was 95.5%. The IPMA assay could be completed in 4 h, compared with more than 6 d for the VNT using rPPRV-GFP, and it is easily performed, as the staining results can be observed under a microscope. Additionally, unlike the VNT, the IPMA does not require antigen purification, which will reduce its cost. In conclusion, the established IPMA will be an alternative method that replaces the VNT for detecting antibodies against PPRV in the field.

## Introduction

Peste des petits ruminants (PPR), caused by the PPR virus (PPRV), is a highly contagious disease[1,2]. The virus belongs to the genus *Morbillivirus*, family *Paramyxoviridae*. Small ruminants such as sheep and goats are the primarily target of PPRV, which has caused huge economic losses to the breeding industry[3]. Clinical symptoms are characterized by ocular and nasal discharge, fever, bronchointerstitial pneumonia, and diarrhea[4,5]. Morbidity and mortality rates can reach 100% and 90%, respectively[6]. PPR is a disease that should be reported to World Organization for Animal Health (OIE). Since December 2013, there have been PPR outbreaks in many regions of China. The prevention and control of PPRV face severe challenges[7], and fast and efficient PPR diagnostic methods are of especially great importance.

It has been demonstrated that the signaling lymphocyte activation molecule (SLAM) is used as a cellular receptor by measles virus, canine distemper virus, rinderpest virus[8–10], and PPRV[11,12]. Histopathologically, morbilliviruses produce large numbers of syncytia in the lymph nodes, splenic white pulp[12], and digestive tract epithelium[13]. After SLAM was identified as the Morbillivirus receptor, SLAM-expressing cell lines, such as Vero-SLAM[14,15], monkey CV1-SLAM[11], human embryonic kidney 293T-SLAM, and Chinese hamster ovary-SLAM[10,16]were established. The characteristic cytopathogenicity produced in SLAM-expressing cell lines after viral infection is similar to that in tissue, and these cell lines have been used for virus isolation and in vitro studies. The established monkey CV1-SLAM cell line expressing goat SLAM is a highly sensitive cell line that is used to isolate PPRV from pathological specimens.

Currently, various methods are used to detect PPRV, such as virus neutralization test (VNT)[17], monoclonal antibody-based complement enzyme-linked immunosorbent assay c-ELISA[18,19], agar diffusion test, hemagglutination tests[20], and virus isolation[21,22], as well as molecular biology-based methods [23–25]. The OIE recommends the VNT and c-ELISA as serologic diagnostic techniques for PPR. The VNT, which has high sensitivity and specificity, is the prescribed test for international trade, but it is laborious, time-consuming. More importantly, the infectious virus was required in VNT[26], which largely restricted the usage in PPRV serological diagnosis[19]. A recombinant N antigen-based c-ELISA is an OIE-approved PPR diagnostic technique that has high relative sensitivity (93.4%) and specificity (98.5%) compared with the VNT reference method[19]. The test is now commercially available (ID Screen® PPR Competition Kit from IDVet, Grabels, France). However, the cost of the test is high because it requires antigen purification and preparation, which are difficult and complicated. Given all these factors, extensive validations of other diagnostic techniques are required.

The immunoperoxidase monolayer assay (IPMA) has many advantages that may help to solve these problems to some extent[27]. First, the preparation of IPMA plates with virus-infected cells is simple, and they can be stored for a long time. Second, IPMA results are examined with an inverted light microscope, which is ideally suited for field conditions[28]. Third, the staining result is stable, and stained specimens can be stored for several months. Based on these advantages, the IPMA has been employed for the rapid diagnosis of many viruses, such as respiratory syncytial virus and influenza virus[29,30], porcine reproductive and respiratory syndrome virus[31,32]and arbovirus[27]. The establishment of IPMA methods involves many factors, of which a suitable cell line is one. Although Vero cells are suitable for the PPRV Nigeria 75/1 strain[33], our study results suggested that the staining result was difficult to observe. Furthermore, goat embryonic kidney and goat testicular cells that can support the reproduction of PPRV are unable to be passaged continuously, which greatly restricts their application. The baby hamster kidney (BHK)-21 cell line expressing goat SLAM has the advantages of rapid growth, the induction of an obvious cytopathic effect (CPE) after infection, and easy observation after staining in an IPMA.

In this study, we aimed to establish a BHK-21 cell line that stably expresses goat SLAM, and to develop an IPMA assay for the diagnosis of PPRV.

## Materials and Methods

### Ethics Statement

The collection of serum samples from the vaccined sheep and goats used in this study was approved by the Animal Care and Use Committee of the Harbin Veterinary Research Institute at the Chinese Academy of Agricultural Sciences in China. The field serum samples came from infected goats or sheep were submitted by owners of the farms for Brucella serum antibody assay, which was also approved by the Animal Care and Use Committee of the Harbin Veterinary Research Institute at the Chinese Academy of Agricultural Sciences in China. The data publication also was approved by owners of the farms.

### Plasmids, cell lines, virus strains and sera

pIRESpuro3 (Clontech, Palo Alto, CA, USA), pCAGG-SLAM-goat, in which the codons of the SLAM gene were optimized using DNASTAR7.0 software according to mammalian codon bias based on the sequence obtained from GenBank (GenBank accession no: DQ228869.1). BHK-21 cells (American Type Culture Collection (ATCC) no.: CCL-10; ATCC, Manassas, VA, USA) were preserved in our laboratory and grown in Dulbecco’s modified Eagle’s medium (DMEM) (Gibco, Grand Island, NY, USA) containing 5% fetal bovine serum (FBS) (Gibco, Grand Island, NY, USA). Vero cells (ATCC no.: CCL-81) were cultured in DMEM containing 10% FBS. All cells are cultured at 37°C in 5% CO2. A recombinant PPRV, based on the PPRV Nigeria 75/1 vaccine strain, expressing the green fluorescent protein (GFP) was generated in our laboratory, named rPPRV/GFP, and titrated on Vero cells[34].

The positive sera samples confirmed with VNT came from vaccinated animals (sheep or goats) were prepared in previous study. Ten sheep and 10 goats were vaccinated with a goat poxvirus-vectored peste-des-petits-ruminants vaccine with a three-weeks interval[35]. Two weeks after the second vaccination, 5 ml bloods per animal were collected and sera were isolated. Eleven goats were vaccinated with PPRV/N75/1, and serum samples were collected four weeks post-vaccination[34].

The sera samples from infected animals were obtained from a farm in Heilongjiang province of China in 2013. Fifty-seven sera samples came from this farm were conducted for *Brucella* serum antibody assay. However, according to the clinical signs and vaccination described by the owners and antibody assay results, all sera samples were positive for PPRV. This farm was determined as PPRV-infected farm.

The negative sera came from another farm in Heilongjiang province of China in 2013–2014 for the *Brucella* serum antibody assay, and the sera samples were also negative for PPRV determined by VNT of PPRV. The source of sera used in this study was listed in Table 1. The sera from PPRV-infected and PPRV-negative farms were originally collected for the *Brucella* serum antibody assay.

**Table 1:** The source and number of sera tested in this study.

Source	Number of sera		Total
	Goat	Sheep	
Vaccinated animals	18	20	38^a^
Infected animals	19	38	57^b^
Negative sera	88	15	103^c^

^a^Sera came from goats and sheep vaccinated with recombinant capripoxvirus (rCPV)

expressing PPRV glycoprotein H (rCPV-PPRV H) or PPRV Nigeria/N75/1.

^b^Field sera came from PPRV-infected farm that had experienced an outbreak of PPRV.

^c^Field sera came from PPRV-negative farm.

### SLAM gene cloning and plasmid construction

According to the optimized SLAM sequence, primers were designed as follows: the upstream primer 5'–TTTGAATTC**GCCGCCACC**ATGGACC–3' contained an EcoRI restriction site (underlined) and Kozak sequence (bold) before the ATG initiation codon to facilitate expression in eukaryotic cells; the downstream primer 5'–TTGCGGCCGCTTA**CTTATCGTCGTCATCCTTGTAATC**GGACTCGGGCACGGTCAC–3' contained a NotI restriction site (underlined) and a FLAG epitope tag sequence (bold). The optimized SLAM gene with a 3' FLAG epitope tag was polymerase chain reaction (PCR)-amplified using the pCAGG-SLAM-goat plasmid as template. The PCR product was double-digested with EcoRI and NotI, and it was subcloned into EcoRI/NotI double-digested pIRESpuro3 plasmid to generate plasmid pIRESpuro3-SLAM.

### Transfection and selection of cell clones

The selection of cell clones was performed as previously described, with some modifications[36,37]. BHK-21 cells were seeded into six-well plates at 0.5×10^6^ cells per well, and grown to 90% confluent. The cells were transfected with pIRESpuro3-SLAM using Lipofectamine 2000 reagent (Invitrogen, Carlsbad, CA, USA) according to the manufacturer’s instructions. Two days after transfection, the cells were passaged (1:30, 1:40 and 1:50 dilutions) in culture dishes and selected in a medium containing 3.0 μg/ml puromycin (Sigma–Aldrich, St. Louis, MO, USA). The medium was changed every 3 d. After 10–14 d of selection, puromycin-resistant cell clones were isolated and cultured in 96-well plates to 90% confluence. The clones were infected with rPPRV/GFP (multiplicity of infection (MOI) = 0.1); cells in which rPPRV/GFP replicated well, as evidenced by many syncytia and a high level of fluorescence, were selected for further single-cell cloning. Finally, the best clones were selected by sequential expansion in 24- and six-well plates, and 25-cm^2^ and 75-cm^2^ flasks, respectively, and the established cell line was named BHK-SLAM.

BHK-SLAM cells were cultured in 75-cm^2^ flasks to 100% confluence. The cells were passaged 1:6 every 2 d. After the 1^st^, 5^th^, 10^th^, 15^th^ and 20^th^ passages, with or without puromycin, the cells were frozen and stored using conventional methods.

### Growth curves

BHK-SLAM cells and Vero cells were seeded into 24-well plates and infected with rPPRV/GFP at an MOI of 0.01. After 1 h of adsorption, the inoculum was removed and the cells were washed twice with DMEM. Then, 0.5 ml of DMEM containing 2% FBS was added to the plates, followed by incubation at 37°C. Viruses isolated at different time points were stored at −70°C until use. The 50% tissue culture infectious dose (TCID_50_) of the virus at different time points was quantified using previously described methods after three freeze-thaw cycles[38].

### Western blotting

BHK-SLAM cells were grown to confluent monolayers in six-well plates. Then, the cells were washed three times with phosphate-buffered saline (PBS) and lysed with radioimmunoprecipitation assay buffer (Solarbio, Beijing, China) according to the manufacturer’s instructions. The lysates were subjected to sodium dodecyl sulfate-polyacrylamide gel electrophoresis, and the proteins were transferred to a nitrocellulose membrane that was blocked (5% non-fat dry milk in PBS) overnight. Then, the nitrocellulose membrane was incubated for 60 min with a mouse monoclonal anti-FLAG antibody (Sigma–Aldrich), followed by Alexa Fluor® 680-conjugated donkey anti-mouse IgG (Sigma–Aldrich) for 60 min, and then the membrane was assayed with an Odyssey infrared fluorescent scanner (Gene Company Limited, Hong Kong, China).

### Preparation of IPMA plates and reaction procedure

BHK-SLAM or Vero cells were grown in 96-well plates to 90% confluence. Cells in columns 1, 3, 5, 7, 9 and 11 were inoculated with rPPRV/GFP at an MOI of 0.01, and cells in columns 2, 4, 6, 8, 10 and 12 were not treated. Then, the plates were incubated at 37°C in 5% CO_2_ for 72 h. After fixing with cold 4% paraformaldehyde for 30 min, the plates were blocked with bovine serum albumen. Before adding serum samples, IPMA plates were blocked again and washed three times with PBS/0.5% Tween-20 (PBST).

Diluted positive and negative PPRV sera were added to the plates, 100 μl/well, and incubated for 60 min at 37°C. After washing three times with PBST, each well was incubated with 100 μl of horseradish peroxidase (HRP)-conjugated mouse anti-sheep IgG (Sigma–Aldrich) for 60 min, 100 μl/well. Fifty microliters of 3-amino-9-ethylcarbazole (AEC) (BD Biosciences, Franklin Lakes, NJ, USA) was added after washing three times with PBST. After a 10-min incubation at 37°C, the plates were washed once with PBS, and the results were observed using an inverted fluorescence microscope (Carl Zeiss AG, Oberkochen, Germany). Wells in the odd numbered columns incubated with negative serum, as well as wells in the even numbered columns, remained unstained, whereas wells in the odd numbered columns incubated with positive serum were stained reddish-brown. The staining and GFP expression results were observed in the same visual field.

### Characterization of the IPMA

To determine the specificity and sensitivity of the IPMA, 198 sera samples were tested, and the results were compared with those of the VNT. The VNT was conducted as previously described[34]. In addition, to test the specificity of the IPMA, 1:10 dilutions of positive sera against PPRV, goat poxvirus (GPV), foot-and-mouth disease virus (FMDV), bluetongue virus (BTV) and *Brucella* were examined to determine their cross-reactions with the PPR-IPMA.

## Results

### Screening and identification of positive cell clones expressing SLAM

**Fig 1:**
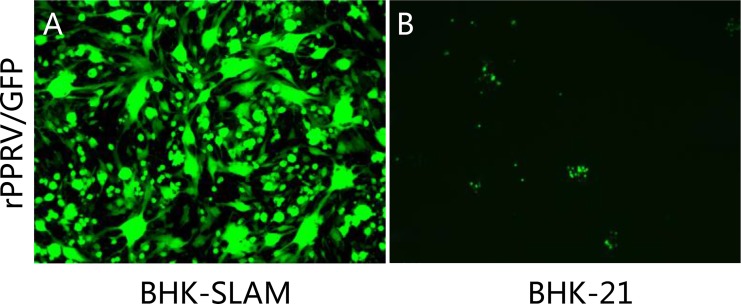
Identification of cell clones expressing SLAM. (A) Puromycin-resistant BHK-21 cell clones and parent BHK-21 cells were infected with rPPRV/GFP at an MOI of 0.1 for 3 d, and puromycin-resistant BHK-21 cells that showed the highest levels of green fluorescent and largest syncytia were selected as high SLAM- expressing cells. (B) The BHK-21 parent cells showed little green fluorescence and no syncytia formation because they did not express SLAM.

BHK-21 cells transfected with the pIRESpuro3-SLAM plasmid were cultured in selection medium containing 3 μg/ml puromycin. Fifty-two surviving cell clones were selected, grown to 90% confluence in 96-well plates, and infected with rPPRV/GFP. BHK-21 cells were used as a control. Three cell clones in which rPPRV/GFP replicated well and that showed many syncytia and high fluorescence levels were selected (Fig 1A), while no syncytia and little fluorescence were observed in BHK-21 control cells (Fig 1B).

### Passage stability of the BHK-SLAM cell line

**Fig 2:**
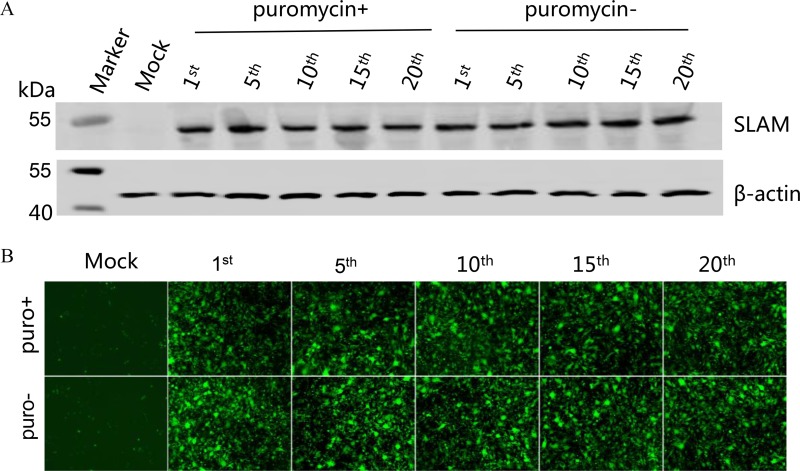
Passage stability of the BHK-SLAM cell line. (A) BHK-SLAM cells of different generations (1^st^, 5^th^, 10^th^, 15^th^, and 20^th^ passages) were cultured, with or without puromycin, to 100% confluence in six-well plates. Then, these cells were collected and prepared for western blotting using an anti-FLAG monoclonal antibody. An approximately 45-kDa band corresponding to the SLAM protein was detected in all of the BHK-SLAM cells of different generations. BHK-21 cells and β-actin (42 kDa) were used as controls. (B) BHK-SLAM cells of different generations (1^st^, 5^th^, 10^th^, 15^th^, and 20^th^ passages), cultured with or without puromycin, were infected with rPPRV/GFP for 3 d, and images were taken. BHK-21 cells were used as a control.

To determine the passage stability of the BHK-SLAM cell line, cells of different generations (1^st^, 5^th^, 10^th^, 15^th^ and 20^th^ passages) were grown to 100% confluence in six-well plates containing medium with or without puromycin. SLAM expression was detected using an anti-FLAG antibody. As a control, β-actin was also detected using an appropriate monoclonal antibody. As shown in Fig 2A, an approximately 45-kDa band corresponding to the SLAM protein was detected in cells of different generations, while a 42-kDa band was observed for β-actin. SLAM expression was not affected by the presence of puromycin. Moreover, regardless of the presence of puromycin, GFP fluorescence and the CPE did not differ among different generations of cells after infection with rPPRV/GFP (Fig 2B).

### BHK-SLAM cells are more appropriate for the IPMA than Vero cells

**Fig 3:**
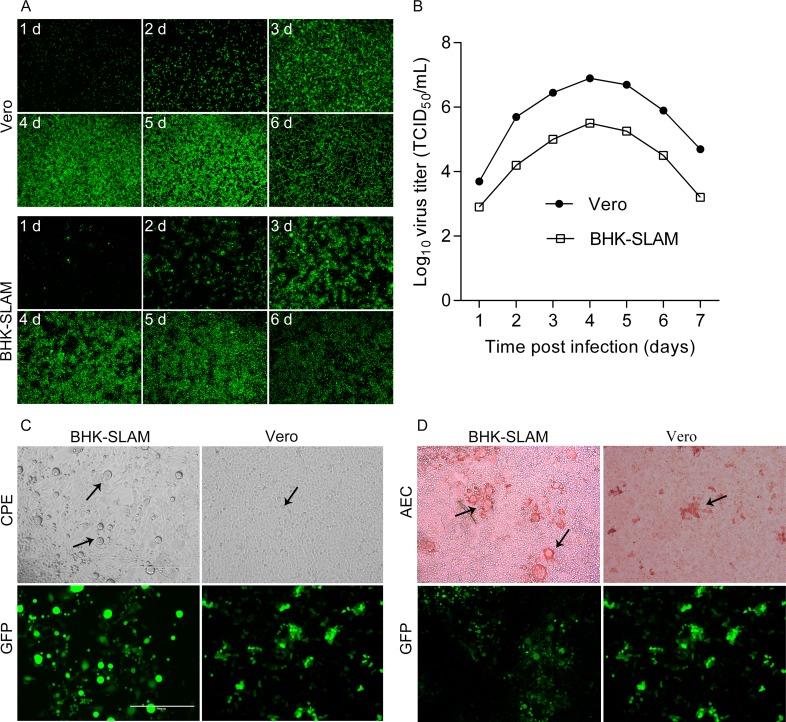
Comparison of BHK-SLAM cells and Vero cells. (A) BHK-SLAM cells and Vero cells were grown to 80%–90% confluence in 24-well plates. Then, they were infected with rPPRV/GFP at an MOI of 0.01. At various time points after infection (every 24 h), images of GFP expression were taken. (B) Viruses at different time points were collected and stored at −70°C, and viral titers (TCID_50_) were determined in Vero cells. (C) BHK-SLAM and Vero cells were fixed at the same time after infection with rPPRV/GFP at an MOI = 0.1. The CPE (black arrow) and GFP expression were observed. (D) After fixing with cold 4% paraformaldehyde for 30 min, the BHK-SLAM and Vero cells were used for the IPMA, and AEC staining results were observed (black arrow).

BHK-SLAM cells were infected with rPPRV/GFP at an MOI of 0.01, and Vero cells were used as a control. The virus replication levels at various time points post virus infection were assessed by analyzing the GFP expression of rPPRV/GFP. As shown in Fig 3A, GFP expression increased until all of the cells were infected 4 d post-infection. The results were consistent with those obtained using Vero cells. Meanwhile, the viral titers were determined in the two cell types, and growth curves were determined (Fig 3B), which showed that the highest titer in BHK-SLAM cells was lower than that in Vero cells. The peak titer in the two cell lines appeared at the same time (4 d post-infection).

The BHK-SLAM and Vero cells were fixed at the same time after infection with the same doses of rPPRV/GFP, and the IPMA was conducted as described above. The results showed that a more obvious CPE and larger syncytia were observed in BHK-SLAM cells, compared with those in Vero cells (Fig 3C). Furthermore, owing to the high growth density of the BHK-SLAM cells and the formation of large syncytia after virus affection, we observed that the AEC staining areas were large and significantly different from those of unstained cells, while these results were not obvious in Vero cells (Fig 3D). As a result, the results of the IPMA were easier to observe than those in Vero cells. Thus, BHK-SLAM cells were used for the IPMA in the following experiments.

### Determination of the optimal reaction conditions of the IPMA

**Fig 4:**
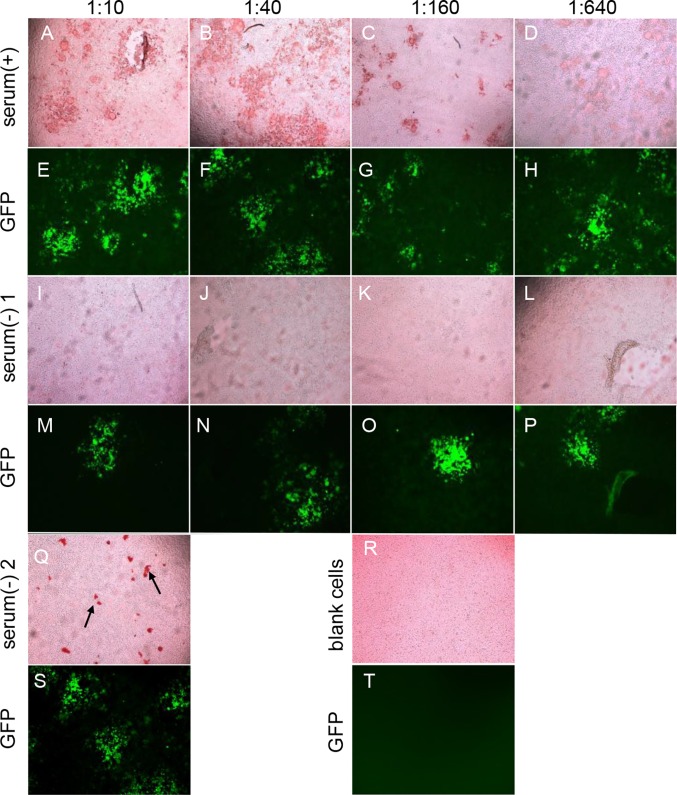
Determination of the optimal reaction conditions of the IPMA. The BHK-SLAM cell line was used in the IPMA. For the IPMA, diluted positive serum (+) (neutralizing titer ≥10) and negative serum (−) (neutralizing titer <10) were added to the test wells, followed by HRP-conjugated mouse anti-sheep IgG, and stained with AEC. The AEC staining images (A–D, I–L) and GFP fluorescence (E–H, M–P) were observed with different sera dilutions (1:10, 1:40, 1:160 and 1:640). A false-positive result was observed for negative serum sample 2, with irregular staining (Q, black arrow), and GFP expression (S) was also observed. Mock-infected cells with no staining (R) and GFP expression (T) were used as a control.

BHK-SLAM cells were infected with rPPRV/GFP at an MOI of 0.01. Then, positive and negative PPRV sera were added to plates at different dilutions (1:10, 1:20, 1:40, 1:80; 1:160, 1:320 and 1:640), followed by 100 μl of HRP-conjugated mouse anti-sheep IgG (diluted 1:5,000). The results for several serum dilutions (1:10, 1:40, 1:160 and 1:640) are shown (Fig 4). Cells that were not inoculated with virus were used as a control. The results indicated that cells incubated with positive sera were stained reddish-brown (Fig 4A–4D), and the staining region was generally similar to that observed for GFP expression (Fig 4E–4H, respectively). When incubated with negative sera, cells that expressed GFP (Fig 4M–4P), as well as mock-infected cells, remained unstained, as shown in Fig 4I–4L and 4R, respectively. With increasing sera dilutions, the positive staining results gradually decreased, but staining was still detected when the serum dilution was 1:640. Considering the low background and obvious staining results, the initial sera dilution of the IPMA was set to 1:10.

However, when some negative serum samples were tested by the IPMA, false-positive results appeared. Irregular staining was observed on the cell surface (Fig 4Q) and corresponding GFP expression was observed (Fig 4S). The GFP expression was regarded as being indicative of the presence of antigens in the IPMA plates; thus, the false-positive results could be eliminated by utilizing GFP expression.

### The established IPMA has high specificity and sensitivity

To test the sensitivity and specificity of the IPMA, the VNT assay was used as a standard, and 198 PPR serum samples from goats or sheep were tested via these methods (Table 2). The results of the VNT showed that there were 95 positive and 103 negative serum samples, whereas there were 86 positive and 103 negative serum samples for the IPMA. There were nine positive sera that were not positively identified by the IPMA. Hence, the IPMA was less specific than the VNT. Compared with the VNT, the sensitivity and specificity of IPMA were 91% and 100%, respectively. The coincidence rate of the two methods was 95.5%.

**Table 2:** Comparison of the IPMA with VNT in 198 serum samples.

	VNT			% agreement^c^	IPMA	
IPMA	No.pos^a^	No.neg^b^	Total		Sensitivity^d^	Specificity^e^
No.pos	86	0	86			
No.neg	9	103	112	95.5	0.91	1
Total	95	103	198			

^a^ No.pos = number of sera with PPRV antibodies.

^b^ No.neg = number of sera without PPRV antibodies.

^c^ (No.pos in both IPMA and VNT) + (No.neg in both IPMA and VNT) / n.

^d^ (No.pos in both IPMA and VNT) / (No.pos in VNT).

^e^ (No.neg in both IPMA and VNT) / (No.neg in VNT).

**Fig 5:**
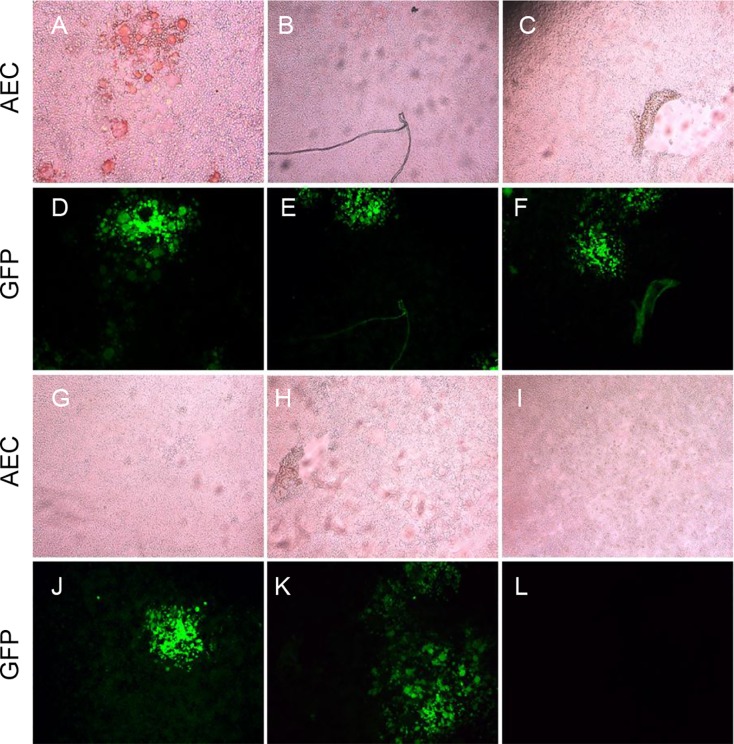
Specificity testing of the IPMA. Panels A–C and G–H show the results of wells reacted with reference sera against PPRV, GPV, FMDV, BTV and *Brucella*. GFP expression is shown in panels D–F and J and K. Panel I shows the result of mock-infected BHK-SLAM cells. The results show that only wells reacted with PPRV sera were stained reddish-brown (panel A).

To test the specificity of the IPMA, sera against PPRV, GPV, FMDV, BTV and *Brucella* were examined using the PPR-IPMA. Only the serum against PPRV resulted in reddish-brown staining (Fig 5A), while other sera against GPV, FMDV, BTV and *Brucella* did not result in any staining (Fig 5B, 5C, 5G and 5H, respectively). The results suggest that there were no serological cross-reactions and confirmed the specificity of the IPMA.

## Discussion

The choice of cells is a critical part of the establishment of an IPMA, as it involves the determination of reaction conditions and the observation of staining results. In this study, we found that Vero cells were not the best choice for a PPR IPMA; thus, we established the BHK-SLAM cell line for the development of the IPMA. This cell line could stably express goat SLAM for at least 20 generations, and we also determined virus growth differences between BHK-SLAM cells and Vero cells after rPPRV/GFP infection. The results indicated that BHK-SLAM cells were able to support virus growth, even though the virus titer was lower than that in Vero cells; the low virus titer in BHK-SLAM cells did not adversely affect the IPMA. Furthermore, BHK-SLAM cells have excellent growth characteristics and can be easily passaged, which are useful for the future commercialization of the IPMA.

Previous studies suggested that SLAM-dependent entry enables PPRV to spread and reproduce efficiently[15,39], which made it easier to isolate the virus while maintaining its antigenicity. Therefore, in this study, we chose goat SLAM to establish the cell line to reduce the possibility of the PPRV Nigeria75/1 strain mutating into new variants. In this way, we ensured the stability of the coated antigen that was used on the IPMA plates. If the BHK-21 cells stably expressing SLAM are not available, the SLAM-transfected BHK cells could be employed, which has the advantages of high transfection efficiency, the induction of an obvious CPE after infection, and easy observation after staining in an IPMA compared with SLAM-transfected Vero cells. However, to confirm the appropriate virus infection does for the preparation of IPMA plates and guarantee the specificity and sensitivity of the test results, BHK-SLAM cells were more suitable than SLAM-transfected BHK cells.

Currently, PPR is prevalent in most of Africa, the Middle East, South Asia, and China[40]. Previous studies indicated that an IPMA has been used to detect sera during outbreaks, which is especially useful when conducting serological studies under field conditions[27]. The IPMA for PPRV diagnosis, which is based on the BHK-SLAM cell line established in the present study, is convenient and reproducible. In contrast with an ELISA, the IPMA does not require highly purified antigen or sophisticated instruments[41], and compared with the VNT, it greatly shortens the diagnosis time. The detection time was only 4 h, which makes the IPMA an adequate substitute for the VNT for assessing herd immunity, as well as for epidemiologic surveillance. The specificity of the IPMA was confirmed by the lack of antibody reactions against other viruses associated with small ruminants. Furthermore, the results of the comparison between the IPMA and VNT, in which 198 serum samples were analyzed, indicated that the IPMA exhibits high specificity and sensitivity. The staining results can be observed directly by an ordinary microscope owing to the huge staining region and low staining background. Thus, this method is fast, cheap, and easy to perform.

Significantly, we used rPPRV/GFP as the coated antigen, and the expression of GFP was useful for the observation of virus infection, which contributed to the quality control of the IPMA plates. GFP utilization in this study helped to eliminate false-positive results, and staining results using BHK-SLAM cells could be observed easily. Thus, the IPMA based on this cell line is an alternative method for detecting PPRV.
